# Relationships Between Spontaneous Alpha Oscillation and Brain Response Amid the Complexity of Brain Adaptation and Spectral Signal Composition

**DOI:** 10.1111/psyp.70087

**Published:** 2025-06-03

**Authors:** Guang Ouyang

**Affiliations:** ^1^ Complex Neural Signals Decoding Lab, Faculty of Education The University of Hong Kong Hong Kong China

## Abstract

The brain operates as a complex dynamic system, continuously generating both structured spontaneous activity and stimulus‐evoked responses. Because these activities originate from the same neural architecture, they are hypothesized to be interconnected. However, research has yet to establish a definitive relationship between spontaneous and response patterns, as findings have been mixed and inconclusive. We argue that this ambiguity stems from significant theoretical and methodological challenges in characterizing the relevant variables amidst the brain's complexity. In this study, we investigated the cross‐individual correlation between spontaneous Alpha amplitude and the magnitude of brain responses to simple stimuli. Our analysis revealed a robust correlation, but only after accounting for two key confounding factors inherent to the brain's complex dynamics: (1) strong adaptation effects across repeated stimulus exposures and (2) the mixture of aperiodic and band‐specific dynamic activity signals. These results demonstrate a close association between the strength of Alpha oscillations—a primary brain rhythm implicated in various functions—and the magnitude of stimuli‐evoked responses. Specifically, individuals with higher resting‐state Alpha amplitudes exhibit stronger brain responses. This discovery not only highlights methodological challenges in relating spontaneous and evoked brain activity, but also demonstrates that they can be addressed. Our findings have significant implications for research aimed at understanding the mechanistic models and functional roles of the brain's dynamic system, shedding light on future investigations into the interplay between intrinsic and evoked neural dynamics.

## Abstract 2

This study reveals a robust correlation between spontaneous Alpha oscillations and brain response magnitude, highlighting the importance of accounting for adaptation effects and aperiodic signal components in EEG analysis. By addressing these methodological challenges, we provide new insights into the brain's dynamic system, demonstrating that individuals with higher resting‐state Alpha amplitudes exhibit stronger responses to stimuli. These findings advance the understanding of neural dynamics and offer a clearer pathway for future research on spontaneous and evoked brain activity.

## 1 Introduction

The dynamical system theory (Deco et al. 2013; Van Gelder 1998; Shapiro 2019; Kelso 1995) is one of the most prominent contemporary frameworks for understanding the inner workings of neural and cognitive systems. At its core, the theory posits that neural systems operate as complex, hierarchical, and self‐sustaining dynamical systems that support cognitive functions at various levels (Van Gelder 1998; Breakspear 2017). Cognitive phenomena emerge from dynamic, interactive processes—whether between functional modules, top‐down and bottom‐up processes, or the brain and body (Shapiro 2019; Kelso 1995). This perspective distinguishes itself from conventional representation and computation theory that likens the cognitive system to a computer or AI system (Shapiro 2019).

From the dynamical system theory, several key relationships between brain signals and cognitive systems can be inferred: First, neural dynamics and their associated signals across different levels or aspects should be interconnected, reflecting the integrative nature of the brain as a dynamical system. One example is cross‐frequency coupling. Second, spontaneous dynamic activities should be linked to stimulus‐evoked responses. Third, the characteristics of the dynamical system are tied to cognitive functions and can serve as markers for individual differences in cognitive performance or ability. Over the past decades, these relationships have been extensively explored, yielding a wealth of supportive evidence across various experimental studies (Spooner et al. 2019; Buzsáki et al. 2012; Lisman and Jensen 2013; Wainio‐Theberge et al. 2021; Ezaki et al. 2020).

To investigate the relationship between spontaneous brain activity and stimulus‐evoked responses, it is essential to characterize and quantify both types of activities from neural signals. However, the heterogeneity of signal composition and the complex variability of brain responses pose significant methodological and conceptual challenges. To elucidate these challenges, we focused on non‐invasive electroencephalography (EEG) in this study, selecting spontaneous Alpha oscillation and event‐related potentials (ERPs) as the representative measures of the two types of activities.

Alpha oscillations were selected due to their dominant presence in neurophysiological signals and their well‐documented functional significance (Sadaghiani and Kleinschmidt 2016). Unlike many other neural signals that are usually obscured by noise, alpha oscillations can be directly observed in raw data—both in time series and spectral representations—without requiring advanced signal processing (Samaha and Romei 2024). Over the past decades, the understanding of Alpha's functional role has evolved considerably, yet a consensus remains elusive. Initially, Alpha oscillations were thought to reflect an idle brain state (Pfurtscheller et al. 1996). However, subsequent studies challenged this view, demonstrating their active involvement in functional processes through both power and phase modulations (Peylo et al. 2021). One influential theory posits that Alpha oscillations represent inhibitory control, with increased Alpha power suppressing task‐irrelevant brain regions (Klimesch et al. 2007). However, other findings also suggest that Alpha reflects a state of tonic alertness, where higher pre‐stimulus Alpha levels enhance response performance (Posner 2008). Collectively, these studies highlight the heterogeneity of Alpha oscillations in both composition and functional roles. Nevertheless, it is well‐established that Alpha, as a prominent spontaneous activity, plays a critical role in shaping brain responses and behavioral performance.

To quantify stimulus‐evoked brain responses, we selected the major component of event‐related potentials: the P3 component, which exhibits a relatively high signal‐to‐noise ratio compared to other stimulus‐evoked components. The P3 is an endogenous brain response observed in ERPs following generic stimuli. Like Alpha oscillations, the P3 component is a heterogeneous complex and has been referred to by various names [e.g., P300, Late Positive Complex, P3 complex; see (Barry et al. 2020)]. This endogenous component is commonly associated with the detection of novelty, context/memory updating, and allocation of attention (Polich 2007; Hajcak and Foti 2020).

Based on the component of Alpha oscillation in the spontaneous EEG activity and the P3 component in the stimulus‐evoked brain response, we aimed to elucidate the issues in the characterization of both amid the complex composition of brain activity signals (elaborated further below). Alongside elucidating these issues, we also report a robust finding regarding the association between Alpha oscillation and brain responses, specifically in the context of cross‐individual variability. This association, derived from a 200‐participant EEG dataset, only becomes apparent after addressing the two methodological challenges.

### 1.1 Issue 1: Characterizing Brain Responses

A common practice in cognitive neuroscience is to measure brain responses by averaging signals across numerous trials to mitigate noise and irrelevant components. While this approach is widely adopted, it has significant limitations, such as the loss of information about trial‐to‐trial variability (Garrett et al. 2013; Stokes and Spaak 2016; Dinstein et al. 2015). A particularly relevant issue for this study is the adaptation effect in brain responses. Given the brain's remarkable capacity for learning and its intrinsic variability across multiple levels of information processing and time scales (McCormick et al. 2020), the response to the first stimulus is fundamentally different from subsequent trials. The first trial, untainted by prior stimuli, reflects a more genuine brain response to a stimulus input without a recent memory in the brain about this input, offering a clearer interaction with task‐free spontaneous activity. In contrast, later trials are influenced by memory and adaptation effects.

In this study, we empirically demonstrated the differences between the first‐trial response and subsequent trials, leveraging data from 200 participants performing a simple visual oddball task. By isolating the first trial, we aim to capture the brain's response in its most unaltered form, free from adaptation effects. This approach allows us to more accurately examine the relationship between resting‐state Alpha amplitude and brain responses. To highlight this difference, we compared two methods of characterizing brain responses: (1) using the first trial alone, and (2) averaging responses across subsequent trials.

### 1.2 Issue 2: Characterizing Neural Oscillations

The second challenge lies in the characterization of neural oscillations. Brain activity spectra consist of both band‐specific oscillations (e.g., Alpha, Beta) and aperiodic components (Ouyang et al. 2020; Wu et al. 2009; Gerster et al. 2022; Donoghue et al. 2020; Miller et al. 2009) [often referred to as fractal activity, scale‐free activity, or 1/f noise (Pei et al. 2023; Brake et al. 2024)]. While the functional significance of these components is increasingly recognized (Klimesch 2012; Yan et al. 2009; Farnes et al. 2020), traditional methods of quantifying oscillations often conflate band‐specific and aperiodic activity. For instance, measuring Alpha amplitude or power without disentangling it from the aperiodic component can lead to misleading conclusions about brain–cognition relationships (Ouyang et al. 2020; Donoghue et al. 2020). Therefore, measuring Alpha amplitude without explicitly correcting for the 1/f component will inherently introduce confounding variance into the Alpha‐band estimates.

### 1.3 Previous Research and Novel Contributions

Previous research on the relationship between spontaneous brain activity and cognitive performance (McCormick et al. 2020; Busch et al. 2009; VanRullen 2016; van Dijk et al. 2008; Mathewson et al. 2009; Romei et al. 2008; Myers et al. 2014) or brain responses (Spooner et al. 2019; Wainio‐Theberge et al. 2021; He 2013; Rajagovindan and Ding 2011) has largely focused on within‐subject, trial‐to‐trial variability. Few studies have explored cross‐individual differences, with notable exceptions such as Dockree et al. (2007), who found a cross‐individual association between Alpha power (however, calculated from task session) and global activation magnitude in ERP, and Spooner et al. (2019), who found that spontaneous activity in the 30–75 Hz range mediates age‐related declines in somatosensory gating. However, the interplay between band‐specific and aperiodic components, as well as the impact of cross‐trial variations (e.g., adaptation), remains underexplored in the context of cross‐individual variability.

This study bridges this gap by investigating the relationship between spontaneous Alpha oscillations and brain responses in a large participant sample. Using ERPs from a visual oddball task, we characterize brain responses while distinguishing between initial and subsequent trials. By mitigating the methodological challenges of signal heterogeneity and intrinsic response variability, we aim to provide new insights into the dynamical system underpinnings of brain activity and cognition.

In summary, this study explores how spontaneous Alpha oscillations relate to brain responses in the context of cross‐individual variability. We demonstrated how methodological issues in signal characterization and response variability can obscure these relationships. Addressing these challenges advances our understanding of the brain's dynamical system and its functional implications.

## 2 Materials and Methods

### 2.1 EEG Dataset and Cognitive Tasks

The EEG dataset was obtained from 200 participants (62 males, 138 females, aged 18–40 years, mean age: 25.1, SD: 4.5) who performed a visual oddball task (Ouyang et al. 2022; Ouyang 2023). The study received approval from the Human Research Ethics Committee (HREC) at the University of Hong Kong (EA1802045). Participants were recruited between January 1, 2019, and June 30, 2021, and all provided written consent prior to the experiment.

Before the task, EEG data were collected during a 2‐min resting state session. Participants were instructed to relax, remain seated, and avoid engaging in intense mental activity. The resting state session consisted of a 60‐s eye‐open period followed by a 60‐s eye‐closed period. During the visual oddball task, participants viewed a sequence of 160 color squares (135 blue, 24 red, 1 yellow) presented individually on a screen for 200 ms each. The task required participants to count the number of different colors in the sequence (the correct answer was three, which was unknown to the participants). The colors blue and red were counterbalanced across participants, meaning that for half of the participants, the sequence consisted of 24 blue, 135 red, and 1 yellow square. The sequence was designed to create stimuli with varying probabilities of occurrence. The color presented 24 times was defined as the “rare” condition (previously referred to as the oddball), while the color presented 135 times was defined as the “frequent” condition. The single yellow square served to inform participants that there were more than just two colors, thereby maintaining their attention. Although the stimulus sequence was pseudo‐random, it was consistent across all participants. The inter‐stimulus interval (ISI) was uniformly distributed between 1700 and 2700 ms.

EEG data were collected in a sound‐attenuated room using Brain Product's actiCHamp amplifier with 32 Ag/AgCl electrode channels, referenced to the amplifier's GND channel connected to the midpoint between Fp1 and Fp2. The 32 channels covered the scalp, and no additional electrooculogram electrodes were used. The channels included: Fp1, Fz, F3, F7, FT9, FC5, FC1, C3, T7, TP9, CP5, CP1, Pz, P3, P7, O1, Oz, O2, P4, P8, TP10, CP6, CP2, Cz, C4, T8, FT10, FC6, FC2, F4, F8, and Fp2. Gel was applied to maintain electrode impedance at or below 10 kΩ before data collection. The online sampling rate was 1000 Hz. The data underwent preprocessing as described in Ouyang (2023) to remove major artifacts. The preprocessing steps included: (i) down‐sampling to 150 Hz; (ii) applying a bandpass filter between 1 and 45 Hz; (iii) identifying problematic electrodes by calculating the standard deviation (SD) of each electrode (electrodes with SDs > 4 median absolute deviations (MADs) were marked as outliers and interpolated using the spherical method in EEGLAB. MAD was defined as: MAD = median(|*A*_*i*_ *—*median(*A*)|), where *A* represents a set of continuous values and *A*_*i*_ denotes each value); (iv) re‐referencing to the average; and (v) applying the extended Infomax Independent Component Analysis (ICA) algorithm in the EEGLAB toolbox (Lee et al. 1999; Delorme and Makeig 2004) to decompose the EEG data into independent source signals corresponding to the number of electrodes. ICA assumes that statistically independent source activities project their signals to the scalp level, with some sources representing artifacts and others originating from neural activity. The decomposition is based on the principle that the inferred source signals are maximally independent. Artifact sources were identified and removed, and the remaining components were back‐projected to the sensor level to create artifact‐free signals. The Multiple Artifact Rejection Algorithm (Winkler et al. 2011) was used to automatically identify non‐neural artifacts at a probability threshold of 0.5.

### 2.2 Characterization of Brain Response and Its Cross‐Trial Variation Based on Event‐Related Potentials (ERP)

Following preprocessing, ERPs were derived from epochs of (−200 ms, 1000 ms), baseline‐corrected based on the window of (−200, 0 ms). To explore the cross‐trial development of brain responses, we leveraged the large sample size of the dataset: single‐trial ERPs were obtained for each participant and averaged across participants based on a matched trial order. We hypothesized that there would be a trend of decreasing energy across trials due to adaptation effects, which would be evident when data from a large number of participants were combined. Since all participants completed 160 trials with identical stimulus content, the temporal order of each trial could be precisely matched across participants, allowing for a clear delineation of systematic cross‐trial variation patterns in the participant‐averaged single‐trial ERPs. The brain response magnitude was calculated as the ERP amplitude from Pz (where the P3 component showed the strongest amplitude in the current data) averaged between 300 and 600 ms covering the main course of P3. Two versions of brain response magnitude were calculated: (1) from a single trial, and (2) from all trials such that the dependence of the relationship between ongoing Alpha and brain response magnitude on the adaptation effects can be examined.

### 2.3 Calculation of Alpha Amplitude From the Resting‐State EEG Spectrum

The EEG spectrum from the resting state was calculated using Bartlett's method (Bartlett 1948). Specifically, a Fourier transform was applied to each 1‐s segment to obtain single‐second spectra (in amplitude mode, not complex values). These spectra were then averaged across all 60 s. This procedure was performed separately for the eye‐open and eye‐closed sessions and for each electrode. The Oz electrode was selected to characterize Alpha oscillation amplitude, as it is located at the center of the spatial distribution of Alpha activity. Alpha amplitude was calculated in two ways to assess the impact of aperiodic background activity in the spectrum. The first method involved averaging the Alpha amplitude from the raw spectrum between 8 and 12 Hz, which included background activity. To eliminate the contribution of background activity, the second method subtracted the average amplitude of the two frequency ranges flanking the Alpha band (3–7 and 13–17 Hz). Specifically, the average amplitudes from 3–7 to 13–17 Hz were calculated. These two averages were then averaged again, and the mean was subtracted from the average Alpha amplitude calculated from 8 to 12 Hz. This simple correction method was chosen over more complex approaches, such as fitting a broad‐band component, to avoid introducing additional variance, which is explained below. The two Alpha amplitude results were termed “raw Alpha” and “corrected Alpha.” To evaluate the stability of Alpha amplitude measurements, the data were split into two halves, and split‐half reliability was calculated using the Spearman–Brown formula. The reliability results were as follows: for raw Alpha, eye‐open: 0.89, eye‐closed: 0.95; for corrected Alpha, eye‐open: 0.93, eye‐closed: 0.94.

### 2.4 Analysis of the Relationship Between the Spontaneous Alpha Amplitude and Brain Response (ERP) Magnitude

We employed two methods to analyze the relationship between Alpha amplitude measured during the resting state and ERP magnitude from the visual oddball task, after correcting for the confounding effects of aperiodic activity underlying the Alpha peak. The first method was a straightforward correlation analysis that correlated the amplitude of corrected Alpha with the ERP magnitude using Pearson correlation. This result was compared with the correlation obtained using raw Alpha amplitude, which was directly measured from the raw spectrum peak.

An alternative method for correcting the aperiodic (or 1/f) component involves model fitting, where both the aperiodic component and oscillations are modeled using parametric functions (Ouyang et al. 2020; Donoghue et al. 2020; Wen and Liu 2016). However, we chose not to use this method due to concerns that imprecise modeling of the aperiodic component across the entire frequency band could introduce additional variance. In model fitting, the aperiodic component is typically modeled as a simple function with a few parameters (e.g., slope and intercept). If the aperiodic spectrum deviates from the assumed function, particularly at the low or high‐frequency ends, it could lead to the injection of variance from distant frequency bands to the Alpha amplitude corrected in this way. For example, if the aperiodic component does not appear as a straight line in log–log space—such as when the right‐end tail (gamma band) tilts upward—the estimation of the straight line will be skewed counter‐clockwise. This skewing effect artificially inflates the estimated amplitude of the aperiodic component in the low‐frequency range overlapping with the Alpha band. Therefore, we opted for a local correction method as described above. Essentially, the local correction method is equivalent to constraining the modeling of the aperiodic component to a much narrower time window (±5 Hz flanking the Alpha band) as a straight line.

To further validate the robustness of our local correction approach, we applied factor analysis as a second method. In this approach, we modeled two orthogonal factors: an Alpha factor and a 1/f factor, to capture cross‐individual variability in the Alpha range (8–12 Hz) and the broader frequency range (3–17 Hz). The amplitudes at 8, 9, 10, 11, and 12 Hz served as indicators for the Alpha factor, while the amplitudes from 3 to 17 Hz served as indicators for the 1/f factor (see Figure 3). This allowed us to separate the variability of the Alpha peak from the underlying aperiodic component. To analyze the relationship between these factors and ERP magnitude, we constructed a structural equation model with ERP amplitude (measured at electrode Pz, averaged from 300 to 600 ms in the first trial) as the dependent variable, and the two factors (Alpha and aperiodic) along with age and gender as independent variables. Given the high correlations between neighboring frequencies estimated by the Fourier transform, noise covariance was allowed for first‐order (e.g., 5 and 6 Hz), second‐order (e.g., 5 and 7 Hz), and third‐order (e.g., 5 and 8 Hz) neighboring frequencies following a previous practice (Ouyang et al. 2020), with some pairs released to ensure model convergence. The overall model architecture is shown in Figure 3, and detailed results are provided in the Supporting Information S1.

## 3 Results

### 3.1 Strong Brain Response Adaptation Over the First Few Trials

**FIGURE 1:**
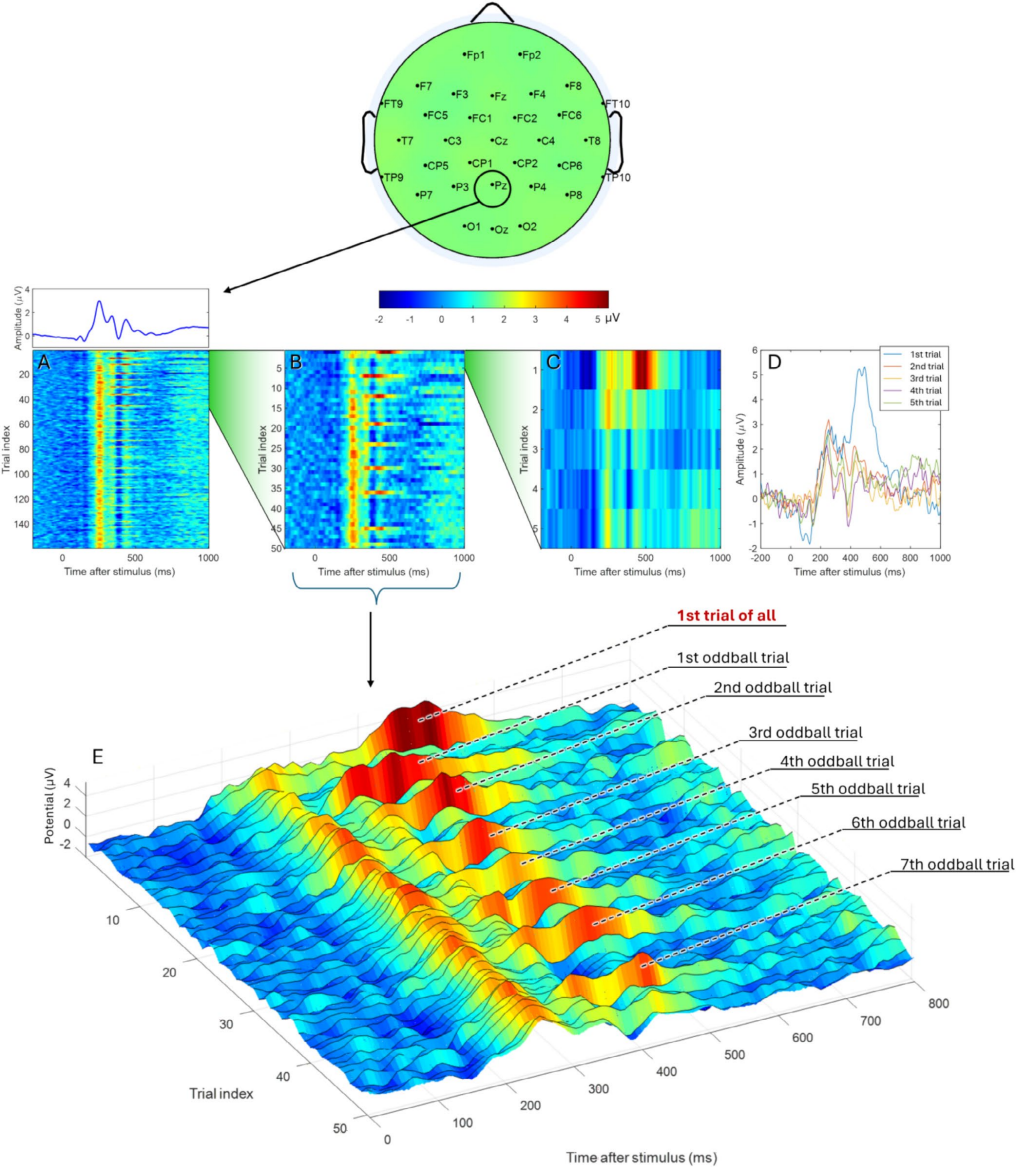
Brain response pattern and its development over trials. The top panel illustrates the electrode layout. (A) Average ERP from electrode Pz, displayed as trial‐average (top) and trial‐to‐trial (bottom) patterns. (B) Zoomed‐in view of A for the first 50 trials. (C, D) Zoomed‐in view of A for the first five trials. (E) Alternative visualization of B.

Figure 1A (top) displays the trial‐averaged ERP waveforms from electrode Pz, further averaged across all 200 participants. Time zero represents the stimulus presentation. Electrode Pz was selected due to its dominant representation of the late positive complex in the current ERP data. The waveform reveals a clear transient dynamic response pattern. In Figure 1A (bottom), single‐trial ERPs are shown, sorted by the temporal order of stimulus presentation (note that each single trial is averaged across 200 participants). The single‐trial patterns (Figure 1A, bottom) align well with the average waveform (Figure 1A, top) in terms of the dynamic unfolding across the first second of the post‐stimulus time window. However, certain cross‐trial development patterns are evident in the single‐trial plot, prompting a closer examination of the first 50 trials (Figure 1B). Figure 1B reveals a conspicuously large amplitude in the first trial, with several large‐amplitude trials scattered throughout the subsequent trials. These large‐amplitude trials (excluding the first trial) correspond to the indices of the oddball trials (i.e., the 7th, 12th, 19th, 24th, 30th, 36th, 45th, etc.), explaining their larger amplitudes. The first trial exhibits the largest amplitude, despite being a frequent (non‐oddball) trial type. This can be attributed to the fact that the first trial is inherently perceived as novel and not yet “frequent” by participants.

Based on the patterns in Figure 1, we propose that the first trial represents a brain response fundamentally distinct from all other trials, including the large‐amplitude oddball trials (Figure 1E). This distinction likely arises because the first trial marks the transition from a pre‐task state to a within‐task state. To further illustrate this, we zoomed in on the first five trials (all of which are frequent trial types). As shown in Figure 1C,D, the first trial exhibits an extraordinarily large amplitude, which rapidly adapts from the second trial onward, demonstrating the brain's strong adaptability, suggesting a transition from spontaneous non‐task state into a task state. A paired t‐test between the first and second trials for ERP amplitude averaged within 300–600 ms at electrode Pz yielded a strong statistical difference: *t* (199) = 3.9, *p* < 0.001. Such between‐trial differences vanished in the subsequent trials: between the second and third trials: *t* (199) = 1.9, *p* = 0.06; between the third and fourth trials: *t* (199) = 0.67, *p* = 0.41; and between the fourth and fifth trials: *t* (199) = −1.2, *p* = 0.22.

### 3.2 Alpha Oscillation and Its Correlation With Brain Response Magnitude

**FIGURE 2:**
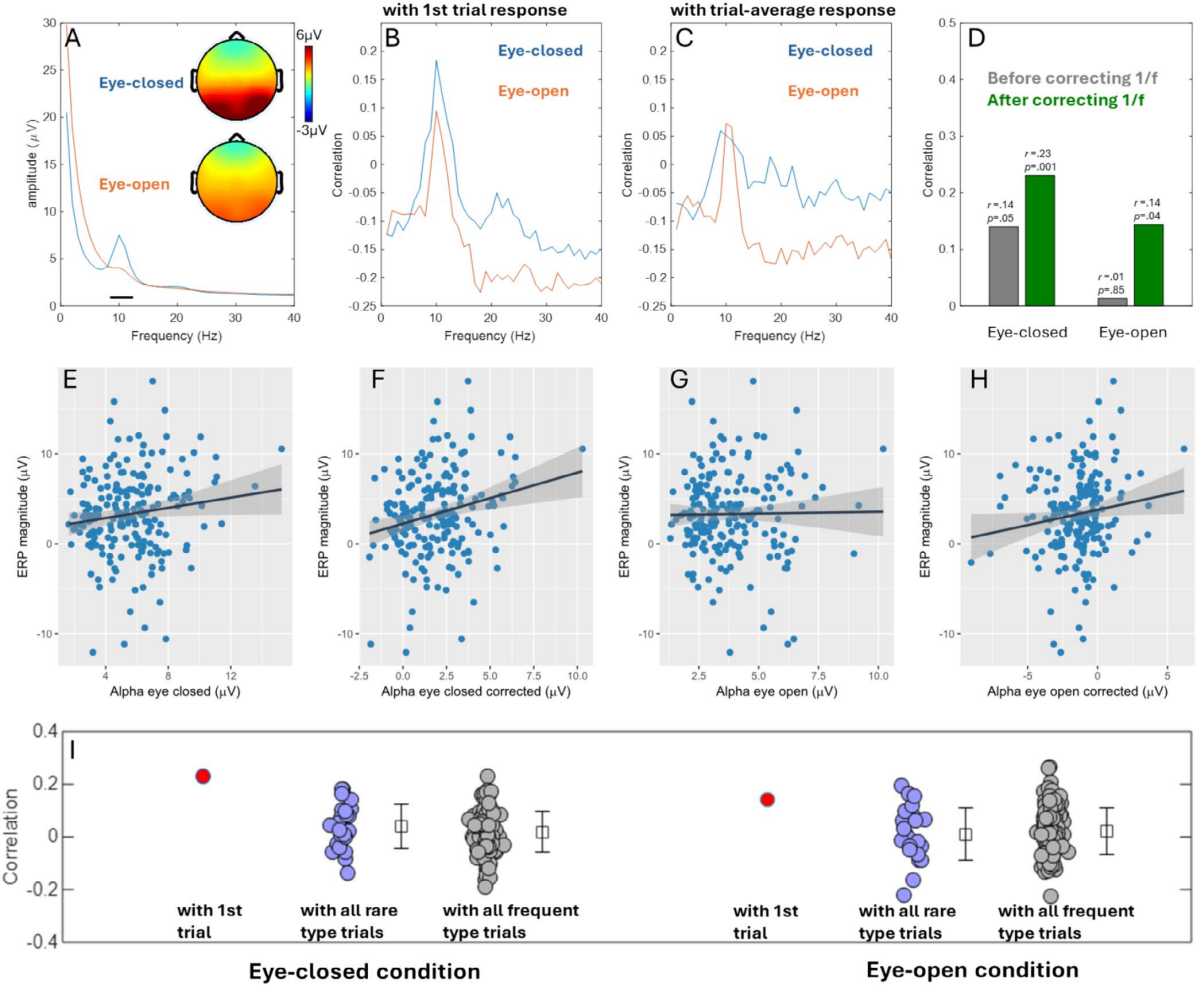
Relationship between Alpha oscillation and brain response magnitude. (A) EEG amplitude spectrum from eye‐open and eye‐closed states. The amplitude distributions within 8–12 Hz are displayed on the scalp maps. (B) Correlation between Alpha amplitude and ERP amplitude from the first trial. The correlation was calculated with a step of 1 Hz. Each value on the integer frequency points represents a correlation coefficient. (C) Same as B, but ERP amplitude is averaged across all trials. (D) Correlation between Alpha amplitude and first‐trial ERP amplitude before and after correcting for the 1/f component in Alpha calculation (note that the correlation here is based on band‐average Alpha, so the value differs from the peak values in B). (E–H) Scatter plots and regression lines for Alpha amplitude (eye‐closed) and ERP magnitude, Alpha amplitude corrected for aperiodic component (eye‐closed) and ERP magnitude, Alpha amplitude (eye‐open) and ERP magnitude, and Alpha amplitude corrected for aperiodic component (eye‐open) and ERP magnitude. The grade shade indicates the standard deviation of the regression line. (I) Correlation between corrected Alpha amplitude from resting state and single trail P3 amplitude, separately presented for 1st trial (red circle on the left), rare type trials (blue circles), and frequent type trials (gray circles). The horizontal jitter of the circles was created for better visualization.

Figure 2A shows the amplitude spectrum from electrode Oz for both eye‐closed and eye‐open resting state sessions. A classic Alpha band peak is evident in both states, though it is much weaker in the eye‐open state. This difference reflects the Berger effect (Berger 1929), where eye‐closing enhances Alpha oscillations in the brain. The scalp distributions of Alpha amplitude (8–12 Hz) for both states are shown in the inset plots, displaying the typical posterior location. Having confirmed the validity of the brain oscillations based on these major characteristics, we proceeded to calculate correlations between resting‐state Alpha oscillation amplitude and brain response magnitude during the oddball task. In an exploratory analysis, we first calculated correlations for all oscillations at different frequencies. We used two versions of brain response: (1) the brain response from the first trial, and (2) the brain response averaged across all trials. The goal was to reveal differences between the first‐trial response and the subsequent trials. Correlations were calculated across participants. Specific parameters were as follows: electrode Oz was selected for oscillation amplitude (as it prominently displays Alpha oscillations); electrode Pz was selected for brain response measurement (as the late ERP is centered at Pz); and the 300–600 ms window was chosen for calculating brain response magnitude, as it covers the peak of the late positive component.

The oscillation‐response correlation results are shown in Figure 2B [first‐trial response and (Figure 2C) trial‐average response]. Two key findings regarding the correlation are highlighted:

1. *Alpha Band Dominance*: The Alpha band exhibits the strongest correlation with brain response magnitude. This is evident in Figure 2B, where a clear peak in the Alpha range aligns with the frequency range of the Alpha peak in the amplitude spectrum (Figure 2A). Importantly, this result is not an artifact of signal processing as there is no theoretical or technical basis for amplitude at a specific given frequency in the spectrum to dictate its correlation with ERP amplitude from a separate task. If this systematic trend existed, Delta and Theta bands would have shown stronger correlations, which is not observed in Figure 2B. Thus, the unique correlation between Alpha oscillations and brain response magnitude reflects a non‐trivial finding.
2. *First‐Trial Dominance*: The Alpha–response correlation is driven primarily by the first‐trial response. This is demonstrated by the difference in correlation values between Figure 2B,C. The distinctive peak pattern in Figure 2B diminishes in the trial‐average ERP (Figure 2C). The overall correlation weakens when later trials are included. This suggests that including subsequent trials (from the second trial onward) obscures the relationship between spontaneous Alpha oscillations in the resting state and brain response to a generic stimulus.

### 3.3 Effect of Aperiodic Brain Activity on the Alpha–Response Relationship

After confirming that the relationship between Alpha oscillations and brain response magnitude is primarily rooted in the first trial, we examined the effect of aperiodic brain activity on this relationship. As is well known, macroscopic brain signals (e.g., EEG) typically exhibit a combination of band‐specific components (e.g., Alpha) and aperiodic components (also termed 1/f or scale‐free activity) in the spectrum. A critical issue is that the aperiodic component contributes significant variance to the measurement of band‐specific oscillation amplitude. This has been highlighted in previous literature and has been shown to potentially mislead conclusions about the functional roles of oscillations (Ouyang et al. 2020; Gerster et al. 2022; Donoghue et al. 2020; Parameshwaran and Thiagarajan 2019).

This issue is also evident in our data. As shown in Figure 2B, although the correlation peak within the Alpha range is conspicuous, the entire correlation curve appears “dragged” downward across all frequencies, reducing the maximum correlation at the peak values (around 10 Hz). This downward drag is hypothesized to result from the confounding effect of aperiodic activity in the EEG spectrum. To address this, we corrected the Alpha amplitude measurement by subtracting the average amplitude of the two frequency ranges flanking the Alpha peak (see Section 2). This correction effectively removes the cross‐individual variability contributed by the underlying aperiodic component.

Figure 2D compares the correlation between Alpha amplitude and ERP magnitude (first trial) before and after correcting for the aperiodic component. After correction, the correlation between eye‐closed Alpha and ERP increased from 0.14 (*p* = 0.05) to 0.23 (*p* = 0.001), and the correlation between eye‐open Alpha and ERP increased from 0.01 (*p* = 0.85) to 0.14 (*p* = 0.04). These results indicate that the aperiodic component counteracts the variability in Alpha amplitude across participants.

Scatter plots for different combinations of Alpha amplitude (corrected/raw, eye‐closed/open) and ERP amplitude are shown in Figure 2E–H. To assess whether gender and age significantly contribute to ERP magnitude variability, we included these factors in the linear models. The statistical results of the four linear regression models are summarized in Table 1. After introducing gender and age, the effect of Alpha in predicting ERP magnitude is consistently stronger in the aperiodic‐corrected version compared to the raw spectrum version, consistent with the results in Figure 2D.

**TABLE 1:** Statistical results of the linear regression models with ERP magnitude as the dependent variable and Alpha amplitude, age, and gender as the independent variables.

	Standardized *β*	*t* statistic	*p*	*R* ^2^	*R* ^2^ adjusted	*F*‐statistic (3, 196)	*p*
Eye‐closed, from raw spectrum							
Alpha	0.14	1.94	0.054	0.052	0.038	3.59	0.015
Gender	−0.05	−0.34	0.073				
Age	−0.18	−2.51	0.013				
Eye‐closed, aperiodic component corrected							
Alpha	0.21	2.92	0.004	0.074	0.060	5.25	0.002
Gender	−0.02	−0.16	0.876				
Age	−0.15	−2.09	0.038				
Eye‐open, from raw spectrum							
Alpha	0.02	0.26	0.797	0.034	0.019	2.32	0.077
Gender	−0.09	−0.61	0.542				
Age	−0.174	−2.46	0.015				
Eye‐open, aperiodic component corrected							
Alpha	0.13	1.76	0.080	0.049	0.034	3.36	0.020
Gender	−0.14	−0.93	0.354				
Age	−0.15	−2.07	0.040				

### 3.4 Addressing Potential Confounding by Spontaneous EEG Activity in First‐Trial ERP Measurements

A potential methodological concern in our prior analysis of the Alpha–response relationship—based on first‐trial ERP measures—is that single‐trial ERP amplitudes may be confounded by the strength of spontaneous, non‐ERP EEG activity. Spontaneous EEG is prominent in both single trials ERP and resting‐state sessions (from which Alpha amplitudes were derived), whereas trial‐averaged ERPs may suppress this activity through averaging. This may explain why the Alpha–response correlation was primarily observed in first‐trial ERP measures rather than in averaged ERPs.

Theoretically, this should not occur: single‐trial P3 amplitudes reflect polarity‐specific evoked activity, while spontaneous EEG fluctuates around zero with no phase locking (and thus should not systematically bias single‐trial ERP estimates). However, to empirically assess this issue, we examined correlations between corrected Alpha amplitude and all single‐trial ERP amplitudes (Figure 2I). The results show that correlation values fluctuate around zero across trials, indicating no systematic bias in single‐trial P3 measures due to shared spontaneous activity between resting‐state EEG and single‐trial ERPs.

Notably, in the eye‐closed condition, the first trial yielded the strongest correlation (*z* = 2.97, *p* = 0.001), while in the eye‐open condition, the first trial remained elevated against the null trend (*z* = 1.62, *p* = 0.052). The zero‐mean fluctuation of the correlation across single trials suggests that the spontaneous Alpha does not systematically confound the correlation, which can be explained by methodological differences: resting‐state Alpha amplitude was derived via Fourier transform (a non‐negative measure of oscillatory amplitude), whereas single‐trial ERP amplitudes were calculated as time‐domain averages over (300–600 ms). Because spontaneous Alpha activity is non‐phase‐locked, individuals with high resting‐state Alpha power need not exhibit high positive ERP amplitudes in a given time window (here, [300, 600 ms]).

### 3.5 Factor Analysis to Examine the Internal Relationships Between Alpha Amplitude, Aperiodic Component, and Brain Response Magnitude

To further validate the differential relationships between Alpha amplitude, the underlying aperiodic component, and brain response magnitude, we applied factor analysis and structural equation modeling (see Section 2). This analysis serves as a triangulation to confirm the cross‐individual association between Alpha amplitude and brain response magnitude, as well as the confounding effect of the aperiodic component on Alpha characterization and the Alpha–response relationship. Instead of directly subtracting the average of the adjacent frequency ranges, we modeled the Alpha band (8–12 Hz) as a single factor and the underlying aperiodic component (3–17 Hz) as another factor. These two factors, along with age and gender as covariates, were used to predict brain response magnitude, which was measured using the first trial. The factor analysis revealed that the relationship between Alpha amplitude and brain response magnitude was consistently significant across eye‐open and eye‐closed conditions (open: *β* = 0.140, *p* = 0.042; closed: *β* = 0.152, *p* = 0.048). The aperiodic factor showed a negative (but non‐significant) correlation with brain response magnitude (open: *β* = −0.07, *p* = 0.37; closed: *β* = −0.04, *p* = 0.56), aligning descriptively with the “downward drag” effect observed in Figure 2B. Full model fitting results are presented in Supporting Information S1.

**FIGURE 3:**
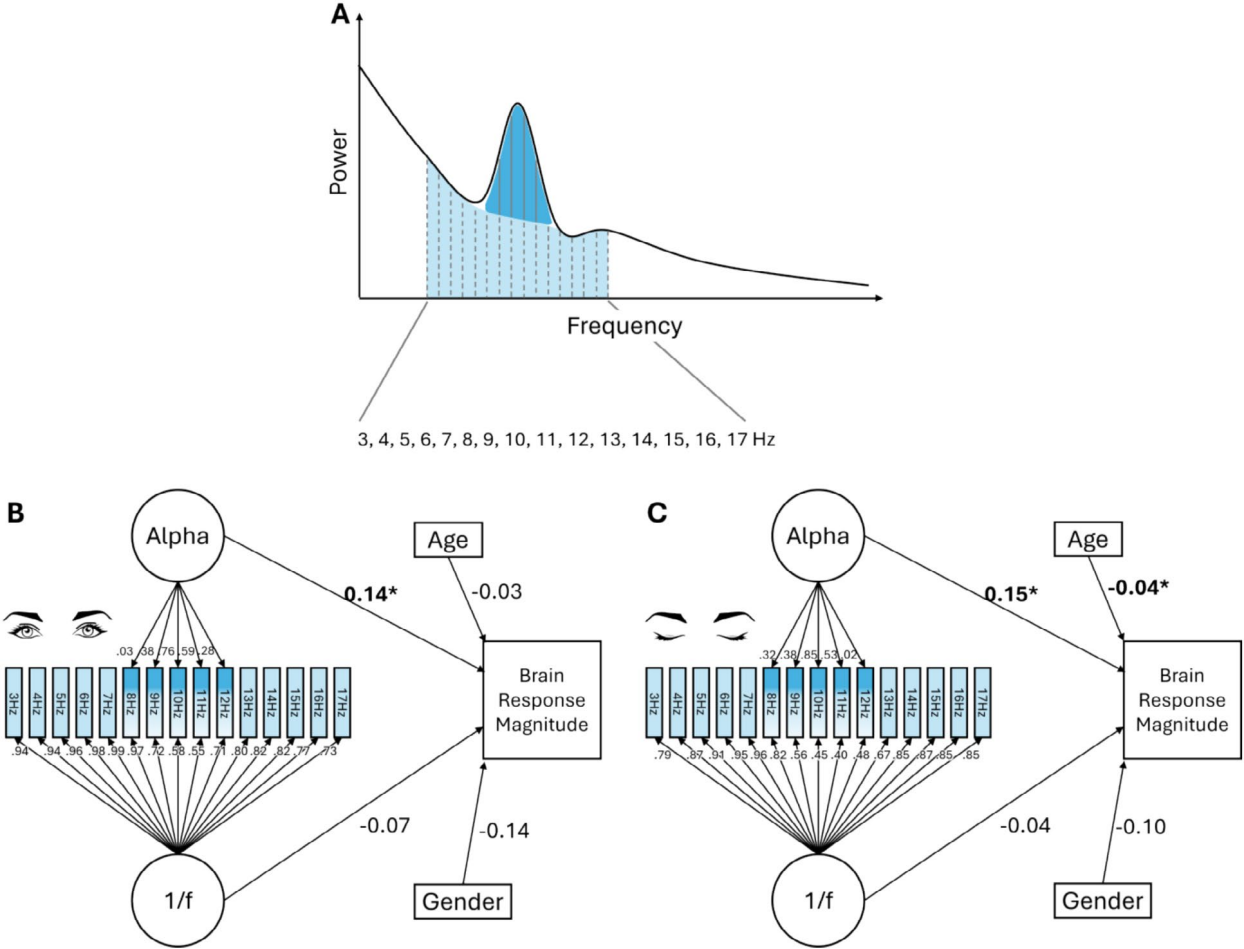
Factor analysis examining the differential relationships between Alpha amplitude, the aperiodic component (1/f), and brain response magnitude. *p < 0.05. For clarity, covariance arrows between the noise of different indicators are not shown. Full model architecture and fit results are shown in Supporting Information S1. (A) Illustration of EEG power spectrum. (B) Model results for eye‐open condition. (C) Model results for eye‐closed condition.

## 4 Discussion

### 4.1 Summary

This study investigated the intrinsic relationship between spontaneous Alpha oscillations and brain response magnitude, explicitly considering the impact of characterization methods on these relationships. Given the well‐documented functional roles of Alpha oscillations' in various neural and cognitive processes as reviewed above, it is reasonable to expect them to influence the brain's response to stimuli. We then selected this relationship as a framework for us to explore relevant methodological and conceptual issues that may complicate the study of spontaneous‐evoked relationships. Our analysis of this relationship focused on the cross‐individual variability, examining how the amplitude of an individual's spontaneous Alpha oscillations affects their brain response magnitude to a generic stimulus. While investigating this relationship at a within‐individual, trial‐to‐trial level would provide additional insights, the current data do not support such an analysis (discussed later). We explored how different characterization methods—grounded in different theoretical assumptions about brain activity and signal properties—might differentially reveal the Alpha–response relationship. Two key factors were considered: (1) the brain's intrinsic adaptation property, which diminishes the response magnitude after the first trial of stimulus exposure, and (2) the mixture of the aperiodic component with pure Alpha oscillations, which introduces variance into Alpha amplitude measurements and obscures the Alpha–response relationship. After accounting for these methodological issues, our results revealed a robust and significant Alpha–response relationship.

### 4.2 Alpha Oscillation and Brain Response

Alpha oscillations are the most prominent and robust oscillations observed in macroscopic brain activity, with extensive functional relevance reported in the literature (Klimesch et al. 2007; Bazanova and Vernon 2014). Unlike other frequency bands, Alpha oscillations exhibit a distinct spectral peak, which is often absent or less pronounced in other bands. As briefly reviewed in Section 1, Alpha oscillations' functional signature has gone through a significant evolution over the past decades, reaching a recent status that both its functional signature and composition are heterogenous. Briefly, Alpha has been linked to a passive role of idle state representation in early views (Pfurtscheller et al. 1996), and active functional roles representing fundamental neural dynamical system property (Breakspear et al. 2006; Freyer et al. 2011), attention (Peylo et al. 2021), alertness (Braboszcz and Delorme 2011), engagement (Bacigalupo and Luck 2022), and general mental intensity (Klimesch et al. 2007). Amidst the heterogeneity, what has been firmly established is Alpha's prominent functional associations. Given this, it is reasonable to expect Alpha oscillations to predict the brain's response to external stimuli. While this relationship has been examined at a trial‐to‐trial level (e.g., Wainio‐Theberge et al. 2021), the cross‐individual relationship based on spontaneous Alpha and brain response measured from *separate* sessions has not been previously reported. Notably, the positive association between spontaneous Alpha and brain response magnitude—both characterized by the *same* task session—has been reported before (Dockree et al. 2007), supporting a positive contribution of Alpha to global mental states such as alertness (Posner 2008). Our current analyses do not just intend to examine the Alpha–response relationship measured from separate sessions, but also to explore how such a relationship is obscured by methodological complexities in characterizing both spontaneous Alpha oscillations and brain responses. Specifically, the characterization of spontaneous Alpha amplitude is affected by the mixture of aperiodic components, and the magnitude of brain responses to a generic stimulus varies significantly between the first and subsequent trials.

Our key finding from the results is that individuals with stronger spontaneous Alpha oscillations tend to exhibit larger brain responses to the first stimulus in the visual oddball task. This result appears to support the view that Alpha is not a simple passive indicator of idleness of the brain, but plays a positive role in general functional status. It aligns well with the alertness view (Posner 2008) that posits that Alpha represents the alertness of mental state when the participants are not engaged in a specific task. As such, individuals with higher Alpha amplitude in the spontaneous brain activity are in a state of being more alert to the incoming stimulus; this also aligns with the fact that Alpha is suppressed during task processing. In contrast, individuals with lower Alpha in the spontaneous activity could be associated with either low alertness to the incoming stimulus or stronger occupation with internal mental activities that occupy their cognitive resources and thus suppress Alpha.

The functional significance of the P3 components accumulated from the previous research also aligns with the Alpha–response relationships observed in this study. P3 is commonly associated with novelty processing and context updating, as supported by its increased magnitude of novel information (Polich 2007; Hajcak and Foti 2020). Assuming that the higher Alpha in the spontaneous EEG signals represents a high state of tonic alertness, our results would suggest that this state intensifies the brain's response to unexpected, novel stimuli. Such unexpected, novel stimuli are best reflected in the first trial as compared to all the subsequent ones, which explains the first trial dominance in the Alpha‐P3 relationships.

While this relationship holds at the cross‐individual level, it should also be observable at the within‐subject, moment‐to‐moment level. That is, for a single participant, higher Alpha amplitude at a given moment should correlate with a larger brain response magnitude. However, testing this requires experimental designs with long inter‐stimulus intervals to ensure genuine spontaneous states and minimal learning or memory effects across trials. Such designs are challenging but necessary for validating the within‐subject Alpha–response relationship.

### 4.3 The Role of Aperiodic Activity (Signal) Component

The aperiodic component of the amplitude spectrum, which exhibits a 1/f‐like pattern, plays a complex role in both theoretical and signal processing aspects of neural cognitive research. This component has been referred to by various names, such as 1/f, 1/f‐like, aperiodic, scale‐free, fractal, broadband activity, or noise. The diversity in terminology reflects unresolved theoretical and conceptual issues related to this component (Buzsáki et al. 2012; Brake et al. 2024; Touboul and Destexhe 2017; Muthukumaraswamy and Liley 2018). In this section, we will discuss two key points related to the aperiodic activity in our current data and analysis.

First, we examined how the presence of the aperiodic signal affects the Alpha–response relationship and how to address this issue. As previously noted, spectral overlap between the aperiodic component and Alpha band introduces variance in Alpha amplitude measurements (Gerster et al. 2022; Donoghue et al. 2020; Muthukumaraswamy and Liley 2018). To mitigate this issue, we employed two approaches in our study.

The first approach involves subtracting the average amplitude of the two adjacent frequency bands from the raw Alpha band amplitude. This method is based on the assumption that the average of the left and right adjacent segments provides an estimate of the aperiodic component underlying the Alpha band, assuming a linear mixture of the Alpha and aperiodic components (Donoghue et al. 2020). While this approach is straightforward, it has limitations, such as potentially introducing Theta–Beta variance into the corrected Alpha amplitude.

To address these limitations, we applied a second approach: factor analysis. This method aimed to capture two latent variables describing the variability of the Alpha and aperiodic components across participants. We then integrated these latent variables into a structural equation model, with brain response magnitude as the dependent variable. The analysis revealed a consistent pattern across both eye‐closed and eye‐open conditions: the Alpha factor consistently predicted brain response magnitude, while the aperiodic factor showed an opposite effect (Figure 3).

It is worth noting that a model‐fitting approach, which models the entire aperiodic component as a parametric function, has been widely used in recent years (Donoghue et al. 2020), including in our previous work (Ouyang et al. 2020; Pei et al. 2023). However, we opted against this method here due to concerns that fitting the wide‐ranging broadband activity as a straight line in logarithmic space might introduce errors in Alpha amplitude estimation. While the scale‐free, 1/f‐like component is often considered a primary feature of complex dynamical systems (Cocchi et al. 2017), solid evidence for this claim is lacking. Most studies report multiple scale‐free components within the aperiodic activity, covering different frequency ranges (Donoghue et al. 2020; Pei et al. 2023; Chaudhuri et al. 2018; Lendner et al. 2020; Kupers et al. 2018), which contradicts the definition of scale‐freeness. Our previous work demonstrated that a single straight line cannot adequately fit the aperiodic component on a log–log scale (Pei et al. 2023), and this multi‐segment pattern has been frequently observed (Miller et al. 2009; Lendner et al. 2020). If the aperiodic activity deviates systematically from the modeled 1/f^β^ pattern in certain frequency ranges (e.g., 0–2 Hz), this deviation can introduce variance into the estimated aperiodic component, which in turn affects the estimated Alpha amplitude. To avoid such systematic errors, we focused on local spectral ranges surrounding the Alpha oscillation.

Second, our data reflects the functional association of the aperiodic component. Despite ongoing theoretical debates about its conceptualization, the functional relevance of the aperiodic component has been widely reported (Ouyang et al. 2020; Lendner et al. 2020; Voytek et al. 2015; Roche et al. 2019). Although we did not specifically model or analyze this component in our study, its functional relevance can be inferred from our descriptive results. As shown in Figure 2B, the aperiodic component exerts a “down‐dragging” effect on the correlation curve, suggesting a negative correlation between the overall amplitude of the aperiodic component and brain response magnitude. However, it remains unclear whether this negative correlation is primarily driven by the slope or the intercept of the 1/f‐like component. If the slope is the primary factor [which is more interesting from a dynamical systems perspective (Gao et al. 2017)], it would imply that individuals with steeper 1/f‐like activity (stronger low‐frequency power and weaker high‐frequency power) generate larger brain responses to generic stimuli. This finding aligns with previous studies linking 1/f‐like activity to functional performance (Ouyang et al. 2020; Pei et al. 2023; Immink et al. 2021; Kasagi et al. 2017), assuming that larger brain response magnitudes are functionally superior—a notion that remains controversial in certain contexts.

## 5 Conclusion and Prospects

Our analysis highlights two significant issues in the study of brain activity and its functional associations. First, the conceptualization and characterization of brain response require careful consideration. While multi‐trial averaging is a common practice, our results demonstrate fundamental differences between first‐trial responses and trial‐averaged responses, particularly in tasks with strong cross‐trial adaptation. This suggests that first‐trial responses may provide more accurate insights in certain contexts. Second, the mixture of oscillatory and aperiodic components in neural signals demands explicit treatment in signal analysis. Our work underscores the importance of addressing this issue to uncover meaningful relationships in neural dynamics.

Finally, our study reveals a significant intrinsic relationship between Alpha oscillations and brain response, offering new insights into the functional significance of Alpha oscillations (Freyer et al. 2011) and the distinct roles of Alpha and aperiodic components in neural cognitive systems (Ouyang et al. 2020; Tröndle et al. 2022). These findings have broad implications for cognitive neuroscience, particularly in understanding the dynamic interplay between neural oscillations and cognitive processes.

## Author Contributions

**Guang Ouyang:** conceptualization, methodology, formal analysis, validation, funding acquisition, writing – original draft, writing – review and editing, investigation.

## Conflicts of Interest

The author declares no conflicts of interest.

## Supporting information


Data S1.

## Data Availability

The datasets and code for the current study will be made available online.
