# Supplementary material for: Relationships Between Spontaneous Alpha Oscillation and Brain Response Amid the Complexity of Brain Adaptation and Spectral Signal Composition
Source: Psychophysiology. 2025 Jun 3;62(6):e70087. doi: 10.1111/psyp.70087 (PMC12134718; doi:10.1111/psyp.70087)
Supplement: Supplementary file 1 — Data S1. [file PSYP-62-e70087-s001.docx]

**Supplementary information**

SEM model fit result for the eye-open condition:

lavaan 0.6.17 ended normally after 320 iterations

Estimator ML

Optimization method NLMINB

Number of model parameters 94

Number of observations 200

Number of missing patterns 1

Model Test User Model:

Test statistic 381.145

Degrees of freedom 90

P-value (Chi-square) 0.000

Model Test Baseline Model:

Test statistic 7555.209

Degrees of freedom 152

P-value 0.000

User Model versus Baseline Model:

Comparative Fit Index (CFI) 0.961

Tucker-Lewis Index (TLI) 0.934

Robust Comparative Fit Index (CFI) 0.961

Robust Tucker-Lewis Index (TLI) 0.934

Loglikelihood and Information Criteria:

Loglikelihood user model (H0) -945.946

Loglikelihood unrestricted model (H1) NA

Akaike (AIC) 2079.892

Bayesian (BIC) 2389.933

Sample-size adjusted Bayesian (SABIC) 2092.132

Root Mean Square Error of Approximation:

RMSEA 0.127

90 Percent confidence interval - lower 0.114

90 Percent confidence interval - upper 0.140

P-value H_0: RMSEA <= 0.050 0.000

P-value H_0: RMSEA >= 0.080 1.000

Robust RMSEA 0.127

90 Percent confidence interval - lower 0.114

90 Percent confidence interval - upper 0.140

P-value H_0: Robust RMSEA <= 0.050 0.000

P-value H_0: Robust RMSEA >= 0.080 1.000

Standardized Root Mean Square Residual:

SRMR 0.093

Parameter Estimates:

Standard errors Standard

Information Observed

Observed information based on Hessian

Latent Variables:

Estimate Std.Err z-value P(>|z|) Std.lv Std.all

f1 =~

alpha_op3 0.939 0.053 17.832 0.000 0.939 0.941

alpha_op4 0.944 0.052 18.077 0.000 0.944 0.949

alpha_op5 0.964 0.051 18.711 0.000 0.964 0.967

alpha_op6 0.982 0.051 19.346 0.000 0.982 0.984

alpha_op7 0.992 0.050 19.690 0.000 0.992 0.994

alpha_op8 0.969 0.051 18.837 0.000 0.969 0.972

alpha_op9 0.720 0.062 11.633 0.000 0.720 0.717

alpha_op10 0.578 0.063 9.103 0.000 0.578 0.590

alpha_op11 0.549 0.061 9.020 0.000 0.549 0.586

alpha_op12 0.711 0.058 12.338 0.000 0.711 0.749

alpha_op13 0.801 0.056 14.393 0.000 0.801 0.833

alpha_op14 0.816 0.052 15.554 0.000 0.816 0.874

alpha_op15 0.818 0.055 14.932 0.000 0.818 0.850

alpha_op16 0.770 0.058 13.366 0.000 0.770 0.789

alpha_op17 0.728 0.061 11.960 0.000 0.728 0.730

f2 =~

alpha_op8 0.030 0.032 0.910 0.363 0.030 0.030

alpha_op9 0.395 0.205 1.931 0.054 0.395 0.394

alpha_op10 0.760 0.141 5.374 0.000 0.760 0.777

alpha_op11 0.592 0.114 5.197 0.000 0.592 0.632

alpha_op12 0.269 0.167 1.615 0.106 0.269 0.284

ERP =~

ERP_pz 0.970 0.048 20.038 0.000 0.994 1.000

Regressions:

Estimate Std.Err z-value P(>|z|) Std.lv Std.all

ERP ~

f1 -0.067 0.074 -0.905 0.365 -0.065 -0.065

f2 0.140 0.069 2.031 0.042 0.136 0.136

ag -0.032 0.016 -1.944 0.052 -0.031 -0.139

gd -0.141 0.156 -0.906 0.365 -0.138 -0.064

Covariances:

Estimate Std.Err z-value P(>|z|) Std.lv Std.all

f1 ~~

f2 0.000 0.000 0.000

.alpha_op3 ~~

.alpha_op4 0.093 0.010 9.159 0.000 0.093 0.881

.alpha_op4 ~~

.alpha_op5 0.070 0.008 8.698 0.000 0.070 0.883

.alpha_op5 ~~

.alpha_op6 0.035 0.006 5.654 0.000 0.035 0.797

.alpha_op6 ~~

.alpha_op7 -0.002 0.006 -0.388 0.698 -0.002 -0.115

.alpha_op7 ~~

.alpha_op8 -0.008 0.008 -0.954 0.340 -0.008 -0.308

.alpha_op8 ~~

.alpha_op9 0.144 0.024 5.896 0.000 0.144 1.069

.alpha_op9 ~~

.alpha_op10 0.123 0.193 0.640 0.522 0.123 0.993

.alpha_op11 ~~

.alpha_op12 0.163 0.115 1.425 0.154 0.163 0.605

.alpha_op12 ~~

.alpha_op13 0.239 0.028 8.569 0.000 0.239 0.792

.alpha_op13 ~~

.alpha_op14 0.169 0.020 8.524 0.000 0.169 0.696

.alpha_op14 ~~

.alpha_op15 0.151 0.016 9.481 0.000 0.151 0.655

.alpha_op15 ~~

.alpha_op16 0.275 0.031 8.976 0.000 0.275 0.906

.alpha_op16 ~~

.alpha_op17 0.369 0.041 9.014 0.000 0.369 0.904

.alpha_op3 ~~

.alpha_op5 0.072 0.009 8.366 0.000 0.072 0.851

.alpha_op4 ~~

.alpha_op6 0.045 0.006 7.274 0.000 0.045 0.820

.alpha_op5 ~~

.alpha_op7 0.004 0.004 0.894 0.371 0.004 0.136

.alpha_op6 ~~

.alpha_op8 -0.004 0.005 -0.823 0.411 -0.004 -0.094

.alpha_op7 ~~

.alpha_op9 0.017 0.011 1.596 0.111 0.017 0.283

.alpha_op8 ~~

.alpha_op10 0.108 0.028 3.905 0.000 0.108 2.148

.alpha_op9 ~~

.alpha_op11 -0.031 0.099 -0.314 0.754 -0.031 -0.114

.alpha_op10 ~~

.alpha_op12 -0.053 0.113 -0.467 0.641 -0.053 -0.432

.alpha_op11 ~~

.alpha_op13 0.142 0.024 5.976 0.000 0.142 0.558

.alpha_op12 ~~

.alpha_op14 0.132 0.020 6.749 0.000 0.132 0.512

.alpha_op13 ~~

.alpha_op15 0.056 0.011 5.078 0.000 0.056 0.207

.alpha_op14 ~~

.alpha_op16 0.146 0.019 7.785 0.000 0.146 0.537

.alpha_op15 ~~

.alpha_op17 0.298 0.034 8.655 0.000 0.298 0.863

.alpha_op3 ~~

.alpha_op6 0.046 0.007 6.993 0.000 0.046 0.776

.alpha_op4 ~~

.alpha_op7 0.010 0.003 3.665 0.000 0.010 0.288

.alpha_op5 ~~

.alpha_op8 -0.005 0.003 -1.799 0.072 -0.005 -0.084

.alpha_op6 ~~

.alpha_op9 0.004 0.005 0.930 0.352 0.004 0.043

.alpha_op7 ~~

.alpha_op10 0.009 0.007 1.226 0.220 0.009 0.387

.alpha_op8 ~~

.alpha_op11 0.039 0.015 2.503 0.012 0.039 0.349

.alpha_op9 ~~

.alpha_op12 -0.068 0.071 -0.964 0.335 -0.068 -0.209

.alpha_op10 ~~

.alpha_op13 0.042 0.014 3.006 0.003 0.042 0.365

.alpha_op11 ~~

.alpha_op14 0.043 0.013 3.212 0.001 0.043 0.197

.alpha_op12 ~~

.alpha_op15 0.023 0.008 2.872 0.004 0.023 0.079

.alpha_op13 ~~

.alpha_op16 0.028 0.008 3.359 0.001 0.028 0.088

.alpha_op14 ~~

.alpha_op17 0.145 0.022 6.636 0.000 0.145 0.466

Intercepts:

Estimate Std.Err z-value P(>|z|) Std.lv Std.all

.alpha_op3 0.000 0.071 0.000 1.000 0.000 0.000

.alpha_op4 0.000 0.070 0.000 1.000 0.000 0.000

.alpha_op5 0.000 0.070 0.000 1.000 0.000 0.000

.alpha_op6 0.000 0.071 0.000 1.000 0.000 0.000

.alpha_op7 0.000 0.071 0.000 1.000 0.000 0.000

.alpha_op8 0.000 0.070 0.000 1.000 0.000 0.000

.alpha_op9 -0.000 0.071 -0.000 1.000 -0.000 -0.000

.alpha_op10 0.000 0.069 0.000 1.000 0.000 0.000

.alpha_op11 0.000 0.066 0.000 1.000 0.000 0.000

.alpha_op12 0.000 0.067 0.000 1.000 0.000 0.000

.alpha_op13 -0.000 0.068 -0.000 1.000 -0.000 -0.000

.alpha_op14 0.000 0.066 0.000 1.000 0.000 0.000

.alpha_op15 0.000 0.068 0.000 1.000 0.000 0.000

.alpha_op16 0.000 0.069 0.000 1.000 0.000 0.000

.alpha_op17 0.000 0.071 0.000 1.000 0.000 0.000

.ERP_pz 0.954 0.420 2.270 0.023 0.954 0.959

Variances:

Estimate Std.Err z-value P(>|z|) Std.lv Std.all

.alpha_op3 0.113 0.012 9.552 0.000 0.113 0.114

.alpha_op4 0.098 0.010 10.001 0.000 0.098 0.099

.alpha_op5 0.064 0.008 7.913 0.000 0.064 0.064

.alpha_op6 0.031 0.007 4.468 0.000 0.031 0.031

.alpha_op7 0.011 0.010 1.175 0.240 0.011 0.011

.alpha_op8 0.054 0.012 4.594 0.000 0.054 0.055

.alpha_op9 0.332 0.163 2.032 0.042 0.332 0.330

.alpha_op10 0.047 0.208 0.223 0.823 0.047 0.049

.alpha_op11 0.227 0.128 1.774 0.076 0.227 0.258

.alpha_op12 0.322 0.094 3.419 0.001 0.322 0.358

.alpha_op13 0.284 0.028 10.177 0.000 0.284 0.307

.alpha_op14 0.207 0.016 12.655 0.000 0.207 0.237

.alpha_op15 0.256 0.027 9.468 0.000 0.256 0.277

.alpha_op16 0.359 0.038 9.451 0.000 0.359 0.377

.alpha_op17 0.465 0.048 9.736 0.000 0.465 0.467

.ERP_pz 0.000 0.000 0.000

f1 1.000 1.000 1.000

f2 1.000 1.000 1.000

.ERP 1.000 0.952 0.952

SEM model fit result for the eye-closed condition:

lavaan 0.6.17 ended normally after 241 iterations

Estimator ML

Optimization method NLMINB

Number of model parameters 94

Number of observations 200

Number of missing patterns 1

Model Test User Model:

Test statistic 310.844

Degrees of freedom 90

P-value (Chi-square) 0.000

Model Test Baseline Model:

Test statistic 5843.049

Degrees of freedom 152

P-value 0.000

User Model versus Baseline Model:

Comparative Fit Index (CFI) 0.961

Tucker-Lewis Index (TLI) 0.934

Robust Comparative Fit Index (CFI) 0.961

Robust Tucker-Lewis Index (TLI) 0.934

Loglikelihood and Information Criteria:

Loglikelihood user model (H0) -1766.876

Loglikelihood unrestricted model (H1) NA

Akaike (AIC) 3721.751

Bayesian (BIC) 4031.793

Sample-size adjusted Bayesian (SABIC) 3733.992

Root Mean Square Error of Approximation:

RMSEA 0.111

90 Percent confidence interval - lower 0.097

90 Percent confidence interval - upper 0.124

P-value H_0: RMSEA <= 0.050 0.000

P-value H_0: RMSEA >= 0.080 1.000

Robust RMSEA 0.111

90 Percent confidence interval - lower 0.097

90 Percent confidence interval - upper 0.124

P-value H_0: Robust RMSEA <= 0.050 0.000

P-value H_0: Robust RMSEA >= 0.080 1.000

Standardized Root Mean Square Residual:

SRMR 0.077

Parameter Estimates:

Standard errors Standard

Information Observed

Observed information based on Hessian

Latent Variables:

Estimate Std.Err z-value P(>|z|) Std.lv Std.all

f1 =~

alpha_cl3 0.790 0.061 12.946 0.000 0.790 0.792

alpha_cl4 0.871 0.057 15.284 0.000 0.871 0.879

alpha_cl5 0.909 0.056 16.301 0.000 0.909 0.915

alpha_cl6 0.947 0.054 17.517 0.000 0.947 0.953

alpha_cl7 0.955 0.054 17.692 0.000 0.955 0.956

alpha_cl8 0.816 0.059 13.810 0.000 0.816 0.821

alpha_cl9 0.555 0.067 8.275 0.000 0.555 0.561

alpha_cl10 0.454 0.069 6.614 0.000 0.454 0.462

alpha_cl11 0.404 0.069 5.817 0.000 0.404 0.418

alpha_cl12 0.482 0.069 6.949 0.000 0.482 0.494

alpha_cl13 0.666 0.065 10.302 0.000 0.666 0.690

alpha_cl14 0.846 0.058 14.705 0.000 0.846 0.879

alpha_cl15 0.871 0.058 15.138 0.000 0.871 0.888

alpha_cl16 0.846 0.060 14.081 0.000 0.846 0.853

alpha_cl17 0.846 0.060 14.063 0.000 0.846 0.848

f2 =~

alpha_cl8 0.321 0.210 1.529 0.126 0.321 0.323

alpha_cl9 0.384 0.279 1.377 0.168 0.384 0.388

alpha_cl10 0.846 0.206 4.104 0.000 0.846 0.862

alpha_cl11 0.526 0.132 3.977 0.000 0.526 0.544

alpha_cl12 0.015 0.049 0.307 0.759 0.015 0.015

ERP =~

ERP_pz 0.968 0.049 19.923 0.000 0.995 1.000

Regressions:

Estimate Std.Err z-value P(>|z|) Std.lv Std.all

ERP ~

f1 -0.043 0.074 -0.587 0.557 -0.042 -0.042

f2 0.152 0.077 1.981 0.048 0.148 0.148

ag -0.036 0.016 -2.208 0.027 -0.035 -0.158

gd -0.097 0.156 -0.618 0.537 -0.094 -0.043

Covariances:

Estimate Std.Err z-value P(>|z|) Std.lv Std.all

f1 ~~

f2 0.000 0.000 0.000

.alpha_cl3 ~~

.alpha_cl4 0.235 0.032 7.317 0.000 0.235 0.817

.alpha_cl4 ~~

.alpha_cl5 0.168 0.024 7.078 0.000 0.168 0.889

.alpha_cl5 ~~

.alpha_cl6 0.085 0.019 4.523 0.000 0.085 0.706

.alpha_cl6 ~~

.alpha_cl7 0.033 0.021 1.585 0.113 0.033 0.376

.alpha_cl7 ~~

.alpha_cl8 0.107 0.023 4.764 0.000 0.107 0.787

.alpha_cl8 ~~

.alpha_cl9 0.283 0.163 1.737 0.082 0.283 0.835

.alpha_cl9 ~~

.alpha_cl10 0.116 0.283 0.408 0.683 0.116 0.773

.alpha_cl11 ~~

.alpha_cl12 0.464 0.061 7.586 0.000 0.464 0.779

.alpha_cl12 ~~

.alpha_cl13 0.479 0.053 9.108 0.000 0.479 0.810

.alpha_cl13 ~~

.alpha_cl14 0.261 0.031 8.463 0.000 0.261 0.812

.alpha_cl14 ~~

.alpha_cl15 0.114 0.021 5.363 0.000 0.114 0.549

.alpha_cl15 ~~

.alpha_cl16 0.191 0.035 5.414 0.000 0.191 0.823

.alpha_cl16 ~~

.alpha_cl17 0.231 0.042 5.453 0.000 0.231 0.847

.alpha_cl3 ~~

.alpha_cl5 0.185 0.028 6.680 0.000 0.185 0.762

.alpha_cl4 ~~

.alpha_cl6 0.097 0.018 5.464 0.000 0.097 0.678

.alpha_cl5 ~~

.alpha_cl7 0.010 0.014 0.715 0.475 0.010 0.087

.alpha_cl6 ~~

.alpha_cl8 0.029 0.013 2.175 0.030 0.029 0.205

.alpha_cl7 ~~

.alpha_cl9 0.154 0.024 6.313 0.000 0.154 0.731

.alpha_cl8 ~~

.alpha_cl10 -0.050 0.212 -0.235 0.814 -0.050 -0.516

.alpha_cl9 ~~

.alpha_cl11 -0.068 0.130 -0.524 0.600 -0.068 -0.134

.alpha_cl10 ~~

.alpha_cl12 0.092 0.046 2.024 0.043 0.092 0.529

.alpha_cl11 ~~

.alpha_cl13 0.275 0.040 6.865 0.000 0.275 0.560

.alpha_cl12 ~~

.alpha_cl14 0.238 0.031 7.757 0.000 0.238 0.613

.alpha_cl13 ~~

.alpha_cl15 0.079 0.015 5.257 0.000 0.079 0.250

.alpha_cl14 ~~

.alpha_cl16 0.090 0.021 4.200 0.000 0.090 0.377

.alpha_cl15 ~~

.alpha_cl17 0.181 0.035 5.116 0.000 0.181 0.761

.alpha_cl3 ~~

.alpha_cl6 0.102 0.018 5.680 0.000 0.102 0.554

.alpha_cl4 ~~

.alpha_cl7 0.012 0.011 1.157 0.247 0.012 0.089

.alpha_cl5 ~~

.alpha_cl8 -0.002 0.005 -0.385 0.700 -0.002 -0.011

.alpha_cl6 ~~

.alpha_cl9 0.039 0.012 3.149 0.002 0.039 0.177

.alpha_cl7 ~~

.alpha_cl10 0.048 0.015 3.108 0.002 0.048 0.791

.alpha_cl8 ~~

.alpha_cl11 -0.083 0.104 -0.796 0.426 -0.083 -0.253

.alpha_cl9 ~~

.alpha_cl12 -0.026 0.016 -1.656 0.098 -0.026 -0.042

.alpha_cl10 ~~

.alpha_cl13 0.047 0.018 2.673 0.008 0.047 0.327

.alpha_cl11 ~~

.alpha_cl14 0.110 0.022 5.034 0.000 0.110 0.339

.alpha_cl12 ~~

.alpha_cl15 0.038 0.012 3.118 0.002 0.038 0.101

.alpha_cl13 ~~

.alpha_cl16 0.023 0.009 2.508 0.012 0.023 0.063

.alpha_cl14 ~~

.alpha_cl17 0.075 0.020 3.672 0.000 0.075 0.307

Intercepts:

Estimate Std.Err z-value P(>|z|) Std.lv Std.all

.alpha_cl3 0.000 0.071 0.000 1.000 0.000 0.000

.alpha_cl4 0.000 0.070 0.000 1.000 0.000 0.000

.alpha_cl5 0.000 0.070 0.000 1.000 0.000 0.000

.alpha_cl6 0.000 0.070 0.000 1.000 0.000 0.000

.alpha_cl7 -0.000 0.071 -0.000 1.000 -0.000 -0.000

.alpha_cl8 -0.000 0.070 -0.000 1.000 -0.000 -0.000

.alpha_cl9 0.000 0.070 0.000 1.000 0.000 0.000

.alpha_cl10 0.000 0.069 0.000 1.000 0.000 0.000

.alpha_cl11 -0.000 0.068 -0.000 1.000 -0.000 -0.000

.alpha_cl12 0.000 0.069 0.000 1.000 0.000 0.000

.alpha_cl13 0.000 0.068 0.000 1.000 0.000 0.000

.alpha_cl14 0.000 0.068 0.000 1.000 0.000 0.000

.alpha_cl15 0.000 0.069 0.000 1.000 0.000 0.000

.alpha_cl16 0.000 0.070 0.000 1.000 0.000 0.000

.alpha_cl17 0.000 0.071 0.000 1.000 0.000 0.000

.ERP_pz 0.999 0.425 2.352 0.019 0.999 1.004

Variances:

Estimate Std.Err z-value P(>|z|) Std.lv Std.all

.alpha_cl3 0.371 0.046 7.986 0.000 0.371 0.372

.alpha_cl4 0.223 0.027 8.295 0.000 0.223 0.227

.alpha_cl5 0.160 0.024 6.793 0.000 0.160 0.162

.alpha_cl6 0.091 0.021 4.345 0.000 0.091 0.092

.alpha_cl7 0.085 0.029 2.905 0.004 0.085 0.085

.alpha_cl8 0.219 0.139 1.571 0.116 0.219 0.222

.alpha_cl9 0.524 0.220 2.378 0.017 0.524 0.535

.alpha_cl10 0.043 0.341 0.125 0.901 0.043 0.044

.alpha_cl11 0.495 0.143 3.472 0.001 0.495 0.529

.alpha_cl12 0.716 0.070 10.267 0.000 0.716 0.755

.alpha_cl13 0.488 0.050 9.664 0.000 0.488 0.524

.alpha_cl14 0.211 0.026 8.190 0.000 0.211 0.228

.alpha_cl15 0.202 0.032 6.399 0.000 0.202 0.211

.alpha_cl16 0.267 0.043 6.222 0.000 0.267 0.272

.alpha_cl17 0.280 0.045 6.235 0.000 0.280 0.281

.ERP_pz 0.000 0.000 0.000

f1 1.000 1.000 1.000

f2 1.000 1.000 1.000

.ERP 1.000 0.948 0.948
